# Awareness of modifiable lifestyle risk factors and acceptability of secondary risk reduction services amongst Irish breast cancer survivors and oncology healthcare professionals

**DOI:** 10.1016/j.breast.2025.104561

**Published:** 2025-08-19

**Authors:** Clara Steele, Janas M. Harrington, Seamus O'Reilly

**Affiliations:** a School of Medicine, University College Cork, Cork, Ireland; b School of Public Health, University College Cork, Cork, Ireland; c Department of Medical Oncology, Cork University Hospital, Cork, Ireland; d Cancer Research @UCC, College of Medicine and Health, University College Cork, Cork, Ireland

**Keywords:** Secondary risk reduction, Lifestyle factors, Breast cancer, Survivorship

## Abstract

Breast cancer is the most common non cutaneous cancer in Irish women. Diagnosis offers a key opportunity to promote lifestyle change. This study assessed awareness of modifiable lifestyle risk factors and acceptability of secondary risk reduction services amongst breast cancer survivors and healthcare professionals.

A cross-sectional study was conducted between September and December 2021. Surveys were developed using previously validated questionnaires: the Mitchelstown Cohort Survey and the International Physical Activity Questionnaire. A survey was offered to healthcare professionals working in and patients attending the early breast cancer outpatient clinic at the South Infirmary Victoria and Cork University Hospitals', Ireland.

322 patients and 29 healthcare professionals participated. Many patients met at-risk lifestyle factors; body mass index >25kg/m2 (66 % [n = 203/313]), low physical activity (19 % [n = 60/322]), frequent consumption of high fat, sugar and salt containing foods (42 % [n = 135/320]), increased alcohol consumption (39 % [n = 125/322]) and current smoker (5 % [n = 17/322]). 83 % of patients and HCPs agreed that modifiable lifestyle risk factors are important in cancer prevention. Only 17 % (n=5/29) of HCPs had training in secondary risk reduction; however, 90 % were willing to refer to services. Patients who had increased alcohol intake or weight gain since diagnosis were more likely to engage with services (44 %, n = 15/34 [p=<0.008]) and (74 %, n = 99/134, [p = <0.001]).

A large proportion of patients met at-risk lifestyle criteria. Gaps in knowledge of at-risk lifestyle behaviours exist. Patients intended engagement with some secondary risk reduction services was associated with their lifestyle behaviours. Our study highlights the challenges of implementing survivorship health promotion programs.

## Highlights

• Modifiable lifestyle factors can increase cancer related morbidity and mortality.
• Irish breast cancer survivors report high awareness of modifiable lifestyle risk factors.
• Many survivors met at risk criteria such as high alcohol use and increased BMI.
• Survivors reported a willingness to engage with secondary risk reductions services.
• Healthcare professionals agree secondary risk reduction is valuable and are willing to refer.

## 1 Introduction

Breast cancer is the most common malignancy in females worldwide [1]. Over the past three decades, improvements in diagnostics and treatments have resulted in 10-year survivorship rates of over 75 % [2,3]. There are over 33,000 breast cancer survivors in Ireland, with incidence expected to rise 130 % by 2040 [2]. Cancer survivorship comes with the risk of disease recurrence, new cancer diagnosis and the development of other non-communicable illnesses, such as cardiovascular disease, the other leading cause of death in our society [4]. Modifiable lifestyle risk factors, including high body mass index (BMI), poor diet quality, low physical activity, high alcohol intake and smoking, are associated with increased risk in many cancer types as well as contributing to recurrence, increased morbidity and mortality [5,6]. In contrast, healthy lifestyles have been associated with better quality of life which in turn is associated with improved prognosis and treatment compliance [[7], [8], [9], [10], [11], [12]].

The Irish National Cancer Strategy (NCS) report 2017–2026 outlines the importance of secondary risk reduction in cancer survivorship. Breast cancer survivors have identified that advice on diet, exercise, and a healthy lifestyle is essential to their survivorship journey however one survey identified that attention to diet and exercise only occurred in slightly more than half of oncology visits [13]. A further survey showed that while oncology providers were attentive to these issues during treatment it often did not result in referral to support services [14]. In Ireland such services are lacking, and clinician engagement is low [15,16]. In contrast, secondary risk reduction services have been successfully implemented in cardiology care for over two decades [17]. Supporting lifestyle modification in large cohorts is complex. Behaviour change requires not only awareness of the need to change but also the modification of environmental factors and a theory-based approach [18]. Moreover, sufficient clinician education, targeted health promotion, awareness of the optimal methods to engage patients, and support from health service providers is crucial [[19], [20], [21]].

The NCS report outlines the importance of the patients' perspective in the development of cancer survivorship services, and this raises research questions on survivors' perspectives. Our aim was to determine the proportion of breast cancer survivors that met at-risk criteria, to understand gaps in awareness and to establish the acceptability of secondary risk reduction services amongst this cohort [5,22]. Additionally, we sought to understand the oncology healthcare professionals’ awareness and acceptability of secondary risk reduction services in clinical practice.

## 2 Methods

### 2.1 Study design

A cross-sectional survey was conducted between September and December 2021 at the South Infirmary Victoria University Hospital and Cork University Hospitals, Ireland. Two surveys were developed using previously validated lifestyle questionnaires [23,24]. Questions on awareness of modifiable lifestyle risk factors and acceptability of secondary risk reduction services were referenced from the NCS guidelines. Surveys were adapted for patients and HCPs accordingly. Questionnaires were distributed to patients attending and HCPs working in the early breast cancer care outpatient service, both a paper and electronic version was offered.

### 2.2 Ethical approval

Ethical approval was granted from the Clinical Research Ethics Committee of The Cork Teaching Hospitals on May 31st, 2021. Data collected was anonymous, with no identifiable information sought. Completion of the questionnaire was regarded as consent to participate. Study data was stored securely in line with GDPR guidelines.

### 2.3 Study measures

Both surveys collected information on participants demographics, lifestyle behaviours and awareness of modifiable risk factors. Participants were asked to comment on intended engagement with five proposed secondary risk reduction services; dietetic-led dietary education and weight management, physiotherapy-led physical education and healthcare professional-led alcohol reduction and smoking cessation services. Questions were presented as Likert scales, checkboxes and open-ended answers.

BMI was categorised into internationally recognised groups [25]. Data on consumption of high-fat, sugar and salt-containing foods was categorised into rare consumers (consumed less than once per week), low regular consumers (1–2 foods consumed 1–4 times per week), high regular consumers (3–4 foods consumed 1–4 times per week), and frequent consumers (foods consumed 5–7 times per week). Fruit and vegetable intake was categorised as meeting none, less than five portions, or five or more portions per day. Physical activity data was scored according to the International Physical Activity Questionnaire Short Form (IPAQ-SF) instructions, and participants were grouped into low, medium, or high activity levels [26]. Excess alcohol intake was defined as greater than six units of alcohol consumed in one sitting. Smoking status was current, former, or never smoker.

### 2.4 Statistical analysis

Statistical analyses were largely descriptive in nature. The distributions of categorial variables were described by counts and percentage in each category, while continuous variables were described by their means and standard deviations, as well as their medians and ranges. Associations between education level and intention to engage with services were estimated using proportional-odds ordinal regression models [27]. Associations between the participants' lifestyle choices and intended engagement were evaluated using Pearson chi-square tests. Statistical analysis was conducted using the R language for statistical computing (Version 4.4.1) [28]. Statistical significance was set at p < 0.05 based on two sided tests.

## 3 Results

### 3.1 Patient demographics & lifestyle behaviours

Three hundred and twenty-two patients (n = 322) completed the questionnaire. All patients identified as female, two-thirds (65 %, n = 209/322) were aged between 51 and 70 years, and 93 % (n = 301/322) reported breast cancer as their first cancer diagnosis (Table 1).

**Table 1:** Patient demographics.

	N = 322^1^
**Age(years)**	
18-30	2/322 (0.6 %)
31-40	19/322 (5.9 %)
41-50	60/322 (19 %)
51-60	135/322 (42 %)
61-70	74/322 (23 %)
71-80	30/322 (9.3 %)
81+	2/322 (0.6 %)
**Gender**	
Female	322/322 (100 %)
Male	0/322 (0 %)
Prefer not to say	0/322 (0 %)
H**ighest level of educationcompleted**	
Primary	20/319 (6.3 %)
Secondary	97/319 (30 %)
Tertiary	202/319 (63 %)
**Diagnosis**	
Breast Cancer (first cancer diagnosis)	301/322 (93 %)
Breast Cancer (second cancer diagnosis)	21/322 (6.5 %)
**First diagnosed with cancer (Year)**	
1990–1999	5/321 (1.6 %)
2000–2009	39/321 (12 %)
2010–2019	207/321 (64 %)
2020-present	70/321 (22 %)
D**iagnosed with more than one cancer**	
Yes	36/322 (11 %)
No	286/322 (89 %)
O**thercancerdiagnosis**	
Breast	14/31 (45 %)
Gastrointestinal	5/31 (16 %)
Lymphoma	5/31 (16 %)
Skin	4/31 (13 %)
Gynaecological	3/31 (9.7 %)
A**pproximate weight(kg)**	73 [16]; 70 [41, 154]; n = 317
**Body Mass Index (kg/m2)**	27.3 (5.8); 26.3 [15.4, 60.2]; n = 313

^1^ n/N (%); Mean (SD); Median [Range]; n = N.

**Fig. 1:**
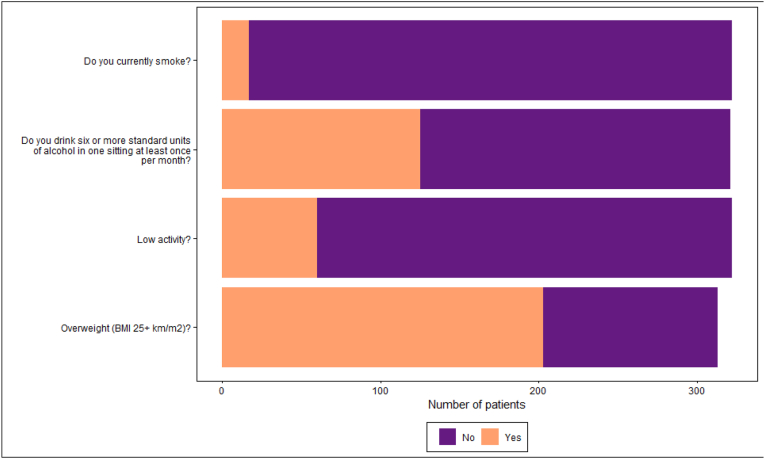
Patients meeting at-risk lifestyle criteria.

Almost two thirds of patients (65 % [n = 203/313]) self-reported a BMI of greater than 25 kg/m^2^ whilst one third (35 % [n = 113/320]) were trying to lose weight (Table 2a, Fig. 1). Forty percent (n = 128/321) followed specific dietary patterns, mostly cholesterol-lowering and weight-reducing, with 6 % (n = 20/321) following a vegan/vegetarian diet. Forty two percent (n = 135/320) frequently or more regularly consumed high-fat, sugar, and salt-containing foods**,** and only 11 % (n = 37/322) reported five or more portions of fruits and vegetables daily. Twenty six percent of patients (n = 85/322) reported stress eating since diagnosis; of these, 9 % (n = 29/322) experienced this daily (Table 2b).

**Table 2a:** Patient anthropometric measurements.

	N = 322^1^
**Body Mass Index category (kg/m^2^)**	
<18.5	3/313 (1.0 %)
18.5 to < 25	107/313 (34 %)
25 to < 30	128/313 (41 %)
30+	75/313 (24 %)
**Trying to lose weight at present**	
Yes	113/320 (35 %)
No	201/320 (63 %)
Unsure	6/320 (2 %)
**Trying to lose weight since diagnosis**	
Yes	132/320 (41 %)
No	181/320 (57 %)
Unsure	7/320 (2.2 %)
**Health professional advised** to **lose, maintain, or gain weight**	
Yes	40/320 (13 %)
No	279/320 (87 %)
Unsure	1/320 (0.3 %)

^1^ n/N (%).

**Table 2b:** Patient dietary habits.

	N = 322^1^
**Dietary patterns**	
None	193/321 (60 %)
Other	11/321 (3.4 %)
Vegetarian	18/321 (5.6 %)
Vegan	2/321 (0.6 %)
Gluten Free	15/321 (4.7 %)
Diabetic	5/321 (1.6 %)
Weight reducing	52/321 (16 %)
Cholesterol lowering	25/321 (7.8 %)
**Portions of fruitconsumed daily**	
0	20/322 (6.2 %)
1 to 4	265/322 (82 %)
5	23/322 (7.1 %)
5+	14/322 (4.3 %)
**Portions of vegetables consumed daily**	
0	6/322 (1.9 %)
1 to 4	271/322 (84 %)
5	21/322 (6.5 %)
5+	24/322 (7.5 %)
**Frequency ofhigh salt, high fat, high sugar and fried foodsconsumed**	
None	1/320 (0.3 %)
Rarely	95/320 (30 %)
Less regularly	89/320 (28 %)
More regularly	86/320 (27 %)
Frequently	49/320 (15 %)
**Stress eating since diagnosis**	
Yes	85/322 (26 %)
No	215/322 (67 %)
Unsure	22/322 (6.8 %)
**Foodintake declined over the last 3 months**	
Yes	65/322 (20 %)
No	248/322 (77 %)
Unsure	9/322 (2.8 %)
**Changes to diet since diagnosis**	
Yes	140/322 (43 %)
No	177/322 (55 %)
Unsure	5/322 (1.6 %)
**Healthcareprofessional discusseddietary change**	
Yes	31/322 (9.6 %)
No	290/322 (90 %)
Unsure	1/322 (0.3 %)

^1^ n/N (%).

According to the IPAQ-SF questionnaire, 19 % (n = 60/322) of patients had a low physical activity level. Forty three percent (n = 139/322) reported not completing any moderate or vigorous activity in the previous seven days, and 2 % (n = 5/322) reported completing no activity in this timeframe (Table 2c, Fig. 1).

**Table 2c:** Patient physical activity.

	N = 322^1^
**Physical activity level (IPAQ)**	
High	123/322 (38 %)
Moderate	139/322 (43 %)
Low	60/322 (19 %)
**Changes tophysical activitysince diagnosis**	
Yes	176/320 (55 %)
No	137/320 (43 %)
Unsure	7/320 (2.2 %)

^1^ n/N (%).

Ten percent of patients (n = 31/322) reported never consuming alcohol and a further 21 % (n = 68/322) reported not drinking alcohol at present. Almost 3 % (n = 9/322) drank 5–6 times per week or more and 39 % (n = 125/322) reported drinking more than six standard units of alcohol in one sitting at least once per month (Table 2d, Fig. 1). Five percent (n = 17/322) were current smokers; of those, 60 % (n = 10/17) smoked every day, and 88 % (n = 15/17) were either thinking of or trying to quit. In the cohort, 43 % (n = 138/322) were former smokers, and 80 % (n = 110/138) of those who previously smoked gave up more than five years ago (Table 2e, Fig. 1).

**Tables 2d:** Patient alcohol consumption.

	N = 322^1^
**Alcohol consumption**	
Not at present	68/322 (21 %)
Never	31/322 (9.6 %)
1–3 times per month	62/322 (19 %)
Once per week	83/322 (26 %)
2–4 times per week	69/322 (21 %)
5–6 times per week	4/322 (1.2 %)
Everyday	5/322 (1.6 %)
**Frequency of consumingsix or more standard units on one occasion**	
At least 1–3 times per month	125/321 (39 %)
Never	102/321 (32 %)
Not at present	94/321 (29 %)
**Changes toalcohol consumption since diagnosis**	
Yes	89/321 (28 %)
No	161/321 (50 %)
Unsure	4/321 (1.2 %)
n/a	67/321 (21 %)
**Health professional discussedalcohol reduction**	
Yes	4/321 (1.2 %)
No	248/321 (77 %)
Unsure	1/321 (0.3 %)
n/a	68/321 (21 %)

^1^ n/N (%).

**Table 2e:** Patient smoking status.

	N = 322^1^
**Smoking status**	
Never Smoked	167/322 (52 %)
Former Smoker	138/322 (43 %)
Current Smoker	17/322 (5.0 %)
**Current smokersdoing any of the following**	
Trying to quit	7/322 (2.2 %)
Actively trying to quit	2/322 (0.6 %)
Not thinking of quitting	2/322 (0.6 %)
Thinking but not planning	6/322 (1.9 %)
**Trying to stop smoking since diagnosis**	
No	8/322 (2.5 %)
Yes	9/322 (2.8 %)
**Health professional discussedsmoking cessation**	
Yes	7/322 (2.2 %)
No	10/322 (3.1 %)
Unsure	0/322 (0 %)
n/a	305/322 (95 %)

^1^ n/N (%).

**Fig. 2:**
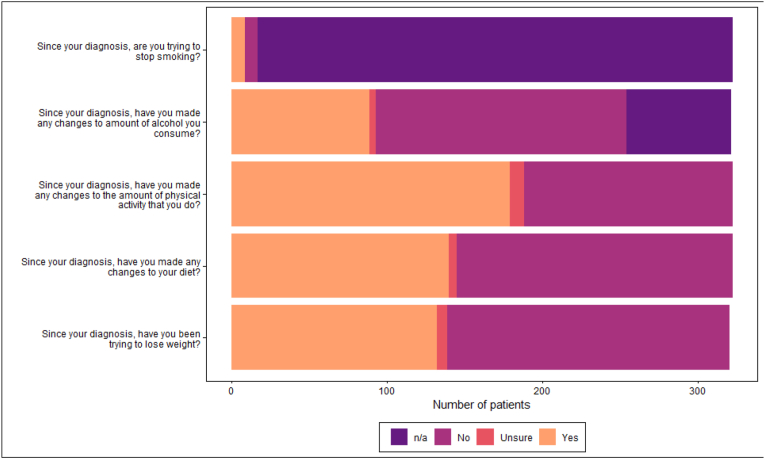
Patient lifestyle changes since diagnosis.

**Fig. 3:**
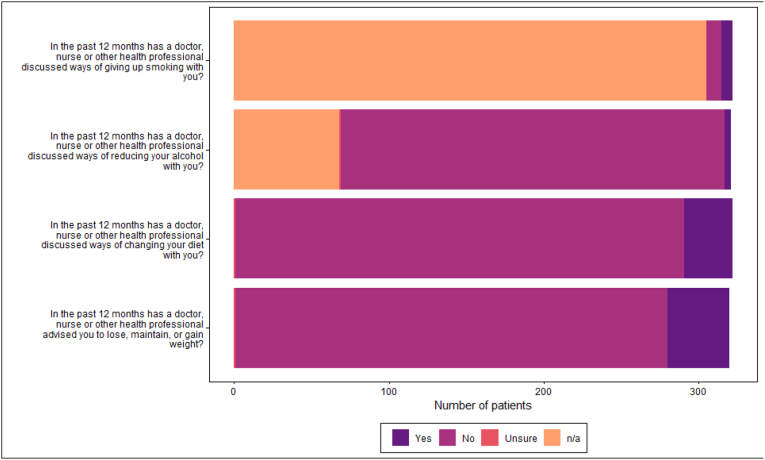
Patients Healthcare provider discussed lifestyle change.

Many patients had made changes to their lifestyle since diagnosis (Fig. 2). HCPs were providing advice on lifestyle changes; however, levels of advice-giving were low compared to the number of patients meeting at-risk criteria (Table 2a, Table 2ea-Table 2e, Fig. 3).

### 3.2 Patient awareness of risk factors and service acceptability

**Fig. 4:**
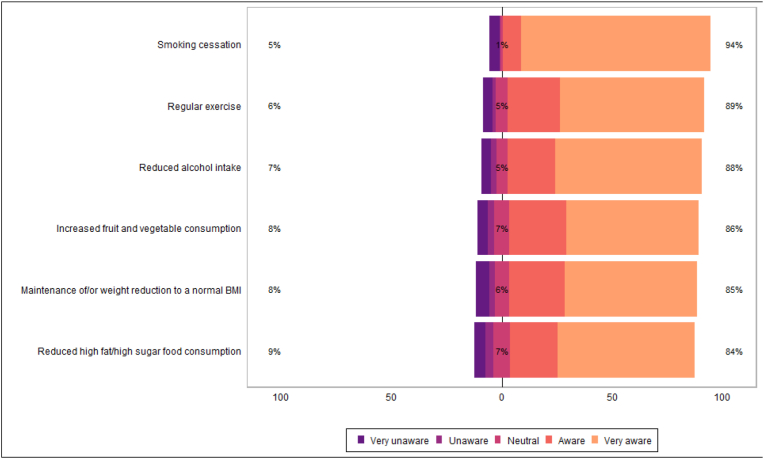
Patient awareness on role lifestyle factors play in cancer development.

**Fig. 5:**
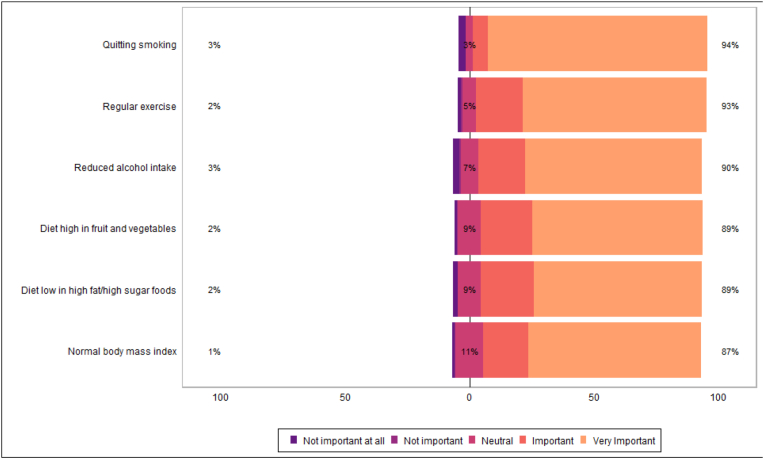
Patient beliefs on importance of lifestyle factors in secondary cancer prevention.

**Fig. 6:**
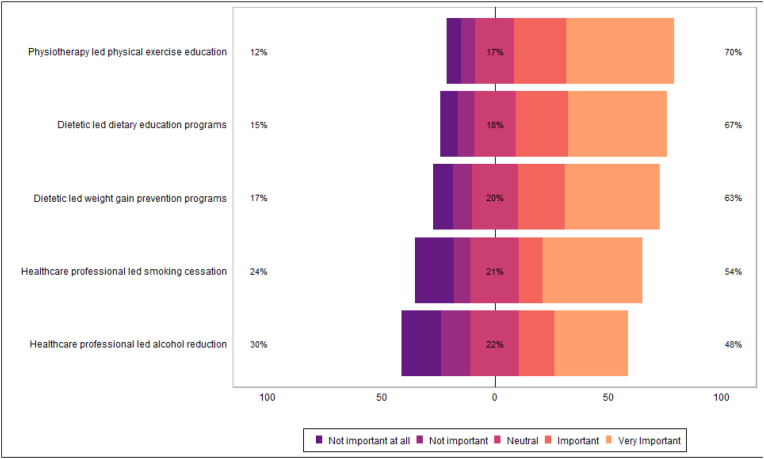
Patient beliefs on importance of access to secondary risk reduction services.

**Fig. 7:**
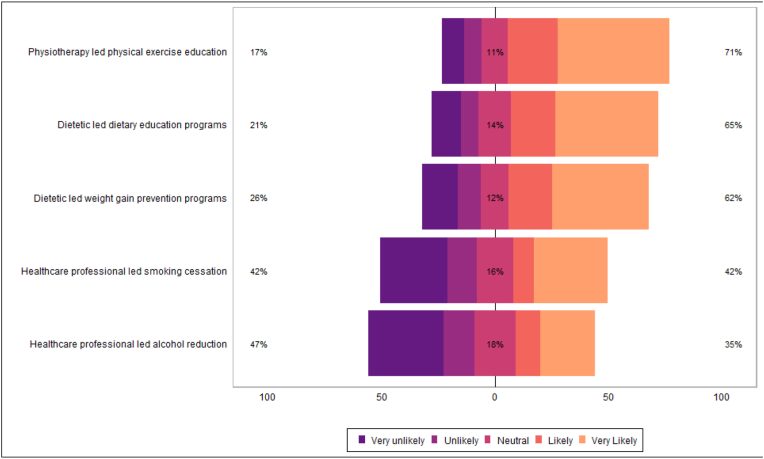
Patient likeliness to engage with secondary risk reduction services.

Patients reported high awareness of lifestyle risk factors in development of primary cancers, and many agreed that lifestyle risk factors were important in secondary cancer prevention (Table 3a, Fig. 4; Table 3b, Fig. 5). Most patients felt that it would be important to have access to weight management, dietary education, and physical exercise education (Table 4a, Fig. 6). Many respondents reported they were likely or very likely to engage with dietetic-led weight management (62 %; n = 166/269), dietetic-led dietary education (65 %; n = 183/281), and physiotherapy-led exercise education services (71 %; n = 200/281), if available (Table 4b, Fig. 7).

**Table 3a:** Patient awareness on role lifestyle factors play in cancer development.

	N = 322^1^
**Maintenance of/or weight reduction to a normal BMI**	
Very unaware	19/312 (6.1 %)
Unaware	7/312 (2.2 %)
Neutral	20/312 (6.4 %)
Aware	79/312 (25 %)
Very aware	187/312 (60 %)
**Increased fruit and vegetable consumption**	
Very unaware	15/315 (4.8 %)
Unaware	9/315 (2.9 %)
Neutral	21/315 (6.7 %)
Aware	82/315 (26 %)
Very aware	188/315 (60 %)
**Reduced high fat/high sugar food consumption**	
Very unaware	16/314 (5.1 %)
Unaware	12/314 (3.8 %)
Neutral	23/314 (7.3 %)
Aware	68/314 (22 %)
Very aware	195/314 (62 %)
**Regular exercise**	
Very unaware	13/314 (4.1 %)
Unaware	5/314 (1.6 %)
Neutral	17/314 (5.4 %)
Aware	74/314 (24 %)
Very aware	205/314 (65 %)
**Reduced alcohol intake**	
Very unaware	12/279 (4.3 %)
Unaware	7/279 (2.5 %)
Neutral	14/279 (5.0 %)
Aware	61/279 (22 %)
Very aware	185/279 (66 %)
**Smoking cessation**	
Very unaware	11/248 (4.4 %)
Unaware	1/248 (0.4 %)
Neutral	3/248 (1.2 %)
Aware	20/248 (8.1 %)
Very aware	213/248 (86 %)

^1^ n/N (%).

**Table 3b:** Patient beliefs on importance of lifestyle factors in secondary cancer prevention.

	N = 322^1^
**Normal body mass index**	
Not important at all	3/319 (0.9 %)
Not important	1/319 (0.3 %)
Neutral	36/319 (11 %)
Important	58/319 (18 %)
Very Important	221/319 (69 %)
**Diet high in fruit and vegetables**	
Not important at all	4/321 (1.2 %)
Not important	1/321 (0.3 %)
Neutral	29/321 (9.0 %)
Important	67/321 (21 %)
Very Important	220/321 (69 %)
**Diet low in high fat/high sugar foods**	
Not important at all	7/321 (2.2 %)
Not important	0/321 (0 %)
Neutral	29/321 (9.0 %)
Important	69/321 (21 %)
Very Important	216/321 (67 %)
**Regular exercise**	
Not important at all	4/320 (1.3 %)
Not important	2/320 (0.6 %)
Neutral	17/320 (5.3 %)
Important	60/320 (19 %)
Very Important	237/320 (74 %)
**Reduced alcohol intake**	
Not important at all	8/315 (2.5 %)
Not important	2/315 (0.6 %)
Neutral	22/315 (7.0 %)
Important	60/315 (19 %)
Very Important	223/315 (71 %)
**Quitting smoking**	
Not important at all	9/308 (2.9 %)
Not important	0/308 (0 %)
Neutral	9/308 (2.9 %)
Important	18/308 (5.8 %)
Very Important	272/308 (88 %)

^1^ n/N (%).

**Table 4a:** Patient beliefs on importance of access to secondary risk reduction services.

	N = 322^1^
**Dietetic led weight gain prevention programs**	
Not important at all	23/266 (8.6 %)
Not important	22/266 (8.3 %)
Neutral	54/266 (20 %)
Important	55/266 (21 %)
Very Important	112/266 (42 %)
**Dietetic led dietary education programs**	
Not important at all	21/269 (7.8 %)
Not important	19/269 (7.1 %)
Neutral	49/269 (18 %)
Important	62/269 (23 %)
Very Important	118/269 (44 %)
**Physiotherapy led physical exercise education**	
Not important at all	17/280 (6.1 %)
Not important	18/280 (6.4 %)
Neutral	48/280 (17 %)
Important	64/280 (23 %)
Very Important	133/280 (48 %)
**Healthcare professional led alcohol reduction**	
Not important at all	36/204 (18 %)
Not important	26/204 (13 %)
Neutral	44/204 (22 %)
Important	32/204 (16 %)
Very Important	66/204 (32 %)
**Healthcare professional led smoking cessation**	
Not important at all	27/160 (17 %)
Not important	12/160 (7.5 %)
Neutral	34/160 (21 %)
Important	17/160 (11 %)
Very Important	70/160 (44 %)

^1^ n/N (%).

**Table 4b:** Patient likeliness to engage with secondary risk reduction services.

	N = 322^1^
**Dietetic led weight gain prevention programs**	
Very unlikely	43/269 (16 %)
Unlikely	27/269 (10 %)
Neutral	33/269 (12 %)
Likely	52/269 (19 %)
Very Likely	114/269 (42 %)
**Dietetic led dietary education programs**	
Very unlikely	36/281 (13 %)
Unlikely	22/281 (7.8 %)
Neutral	40/281 (14 %)
Likely	55/281 (20 %)
Very Likely	128/281 (46 %)
**Physiotherapy led physical exercise education**	
Very unlikely	27/281 (9.6 %)
Unlikely	22/281 (7.8 %)
Neutral	32/281 (11 %)
Likely	62/281 (22 %)
Very Likely	138/281 (49 %)
**Healthcare professional led alcohol reduction**	
Very unlikely	58/176 (33 %)
Unlikely	24/176 (14 %)
Neutral	32/176 (18 %)
Likely	19/176 (11 %)
Very Likely	43/176 (24 %)
**Healthcare professional led smoking cessation**	
Very unlikely	35/118 (30 %)
Unlikely	15/118 (13 %)
Neutral	19/118 (16 %)
Likely	11/118 (9.3 %)
Very Likely	38/118 (32 %)

^1^ n/N (%).

Education level was not associated with self-reported intended engagement with services; weight gain prevention *(p = 0.53),* dietary education *(p = 0.45),* physical exercise education *(p = 0.08),* alcohol reduction *(p = 0.21*) or smoking cessation *(p = 0.74)* (Table 5). Patients who reported weight gain since diagnosis were more likely to engage with weight management services (74 %, n = 99/134, [p = <0.001]) (Table 6a). Patients who drank greater than six units of alcohol at least once per month were more likely to engage with alcohol reduction services (44 %, n = 15/34 [p=<0.008]) (Table 6b).

**Table 5:** Patient likeliness to engage with secondary risk reduction services and education level completed.

	Education level completed			p-value^2^
	Primary, N = 20^1^	Secondary, N = 97^1^	Tertiary, N = 202^1^	
**Dietetic led weight gain prevention programs**				**0.53**
Very unlikely	2/17 (12 %)	7/79 (8.9 %)	34/172 (20 %)	
Unlikely	0/17 (0 %)	17/79 (22 %)	10/172 (5.8 %)	
Neutral	2/17 (12 %)	13/79 (16 %)	18/172 (10 %)	
Likely	5/17 (29 %)	12/79 (15 %)	35/172 (20 %)	
Very Likely	8/17 (47 %)	30/79 (38 %)	75/172 (44 %)	
Unknown	3	18	30	
**Dietetic led dietary education programs**				**0.45**
Very unlikely	1/19 (5.3 %)	7/82 (8.5 %)	28/179 (16 %)	
Unlikely	0/19 (0 %)	13/82 (16 %)	9/179 (5.0 %)	
Neutral	5/19 (26 %)	12/82 (15 %)	23/179 (13 %)	
Likely	3/19 (16 %)	18/82 (22 %)	34/179 (19 %)	
Very Likely	10/19 (53 %)	32/82 (39 %)	85/179 (47 %)	
Unknown	1	15	23	
**Physiotherapy led physical exercise education**				**0.08**
Very unlikely	1/19 (5.3 %)	7/81 (8.6 %)	19/180 (11 %)	
Unlikely	2/19 (11 %)	11/81 (14 %)	9/180 (5.0 %)	
Neutral	3/19 (16 %)	11/81 (14 %)	18/180 (10 %)	
Likely	3/19 (16 %)	22/81 (27 %)	37/180 (21 %)	
Very Likely	10/19 (53 %)	30/81 (37 %)	97/180 (54 %)	
Unknown	1	16	22	
**Healthcare professional led alcohol reduction**				**0.21**
Very unlikely	3/12 (25 %)	12/51 (24 %)	43/112 (38 %)	
Unlikely	0/12 (0 %)	10/51 (20 %)	14/112 (13 %)	
Neutral	2/12 (17 %)	12/51 (24 %)	18/112 (16 %)	
Likely	3/12 (25 %)	4/51 (7.8 %)	12/112 (11 %)	
Very Likely	4/12 (33 %)	13/51 (25 %)	25/112 (22 %)	
Unknown	8	46	90	
**Healthcare professional led smoking cessation**				**0.74**
Very unlikely	3/9 (33 %)	8/41 (20 %)	24/67 (36 %)	
Unlikely	0/9 (0 %)	9/41 (22 %)	6/67 (9.0 %)	
Neutral	1/9 (11 %)	8/41 (20 %)	10/67 (15 %)	
Likely	1/9 (11 %)	5/41 (12 %)	5/67 (7.5 %)	
Very Likely	4/9 (44 %)	11/41 (27 %)	22/67 (33 %)	
Unknown	11	56	135	

^1^ n/N (%).

^2^ p-value based on ordinal regression of intent on education level.

**Table 6:** Patient health behaviours and likeliness to enage with secondary risk reduction services.

Table 6a	Weight gain since diagnosis			p-value^2^
	Yes, N = 156^1^	No, N = 162^1^	Unsure, N = 4^1^	
**Dietetic led weight gain prevention programs**				**<0.001**
Likely	99/134 (74 %)	65/131 (50 %)	2/4 (50 %)	
Not likely	35/134 (26 %)	66/131 (50 %)	2/4 (50 %)	
Unknown	22	31	0	

Table 6b	Frequency of greater than six units of alcohol in one sitting							p-value^2^
	Never, N = 102^1^	Not at present, N = 94^1^	1-3 times per month, N = 52^1^	Once per week, N = 46^1^	2-4 times per week, N = 26^1^	5-6 times per week, N = 0^1^	Every day, N = 1^1^	
**Healthcare professional led alcohol reduction**								**0.008**
Likely	10/52 (19 %)	11/35 (31 %)	15/34 (44 %)	13/34 (38 %)	13/21 (62 %)	0/0 (NA%)	0/0 (NA%)	
Not likely	42/52 (81 %)	24/35 (69 %)	19/34 (56 %)	21/34 (62 %)	8/21 (38 %)	0/0 (NA%)	0/0 (NA%)	
Unknown	50	59	18	12	5	0	1	

^1^ n/N (%).

^2^ Pearson's Chi-squared test.

### 3.3 Healthcare professional demographics & lifestyle behaviours

Twenty-nine HCPs (n = 29) completed the questionnaire; 62 % (n = 18/29) were aged between 31 and 50 years, 66 % (n = 19/29) identified as female, and 17 % (n = 5/29) had postgraduate training in secondary prevention. Of the respondents, 52 % (n = 15/29) were doctors, 24 % (n = 7/29) were clinical nurse specialists and 24 % (n = 7/29) included other allied health professionals (Table 7).

**Table 7:** Healthcare professional demographics.

	N = 29^*1*^
**Age (years)**	
18-30	5/29 (17 %)
31-40	11/29 (38 %)
41-50	7/29 (24 %)
51-60	5/29 (17 %)
61-70	1/29 (3.4 %)
71-80	0/29 (0 %)
81+	0/29 (0 %)
**Gender**	
Female	19/29 (66 %)
Male	10/29 (34 %)
Prefer not to say	0/29 (0 %)
**Occupation**	
Clinical Nurse Specialist	7/29 (24 %)
Registrar	8/29 (28 %)
Specialist Registrar	5/29 (17 %)
Consultant	2/29 (6.9 %)
Other	7/29 (24 %)
**Oncology experience(Years)**	
<1	4/28 (14 %)
1-4	13/28 (46 %)
5-9	6/28 (21 %)
>10	1/28 (3.6 %)
+20	4/28 (14 %)
**Completed postgraduate training in secondary prevention**	
Yes	5/29 (17 %)
No	23/29 (79 %)
Prefer not to say	1/29 (3.4 %)
**Approximate weight (kg)**	77 [18]; 75 [51, 120]; n = 28
**Body Mass Index (kg/m^2^)**	25.1 (6.6); 25.4 [0.0, 38.3]; n = 29

^*1*^ n/N (%); Mean (SD); Median [Range]; n = N.

Forty five percent of HCPs (n = 13/29) were following specific dietary habits, mainly weight-reducing (21 %, n = 6/29) or a vegan/vegetarian diet (17 %, n = 5/29). HCPs met the following at-risk criteria; BMI >25kg/m2 (59 %, n = 17/29), frequent or more regular consumption of high fat, salt, and sugar-containing foods (52 %, n = 15/29), and over half reported taking less than five portions of fruits and vegetables daily. Forty one percent (41 %, n = 12/29) reported stress eating (Table 8a, Table 8b, Table 8c, Table 8d, Table 8ea–e).

**Table 8a:** Healthcare professional anthropometic measurements.

	N = 29^1^
**Body Mass Index category (kg/m^2^)**	
< 18.5	2/29 (6.9 %)
18.5 to < 25	10/29 (34 %)
25 to < 30	12/29 (41 %)
30+	5/29 (17 %)
**Overweight (BMI 25+ kg/m2)**	
No	12/29 (41 %)
Yes	17/29 (59 %)
**Trying to lose weight at present**	
Yes	12/29 (41 %)
No	17/29 (59 %)
Unsure	0/29 (0 %)
**Clinical exposure resulted in changes to lifestyle choices (Weight maintenance/reduction to a normal BMI)**	
n/a	2/29 (6.9 %)
Strongly Disagree	3/29 (10 %)
Disagree	6/29 (21 %)
Neutral	4/29 (14 %)
Agree	12/29 (41 %)
Strongly Agree	2/29 (6.9 %)

^*1*^ n/N (%).

**Table 8b:** Healthcare professional dietary habits.

	N = 29^*1*^
**Dietary patterns**	
None	15/29 (52 %)
Other	1/29 (3.4 %)
Vegetarian	5/29 (17 %)
Vegan	0/29 (0 %)
Gluten Free	1/29 (3.4 %)
Diabetic	1/29 (3.4 %)
Weight reducing	6/29 (21 %)
Cholesterol lowering	0/29 (0 %)
**Frequency of high salt, high fat, high sugar and fried foods consumed**	
None	0/29 (0 %)
Rarely	4/29 (14 %)
Less regularly	10/29 (34 %)
More regularly	11/29 (38 %)
Frequently	4/29 (14 %)
**Clinical exposure resulted in changes to own lifestyle choices (Reduced high fat/high sugar food consumption)**	
n/a	1/29 (3.4 %)
Strongly Disagree	4/29 (14 %)
Disagree	2/29 (6.9 %)
Neutral	6/29 (21 %)
Agree	14/29 (48 %)
Strongly Agree	2/29 (6.9 %)
**Portions of fruit consumed daily**	
<5	17/20 (85 %)
5+	3/20 (15 %)
**Portions of vegetables consumed daily**	
0	2/18 (11 %)
<5	13/18 (72 %)
5+	3/18 (16.6 %)
**Clinical exposure resulted in changes to own lifestyle choices (Increased fruit and vegetable consumption)**	
n/a	1/29 (3.4 %)
Strongly Disagree	3/29 (10 %)
Disagree	4/29 (14 %)
Neutral	3/29 (10 %)
Agree	14/29 (48 %)
Strongly Agree	4/29 (14 %)
**Stress eating**	
Yes	12/29 (41 %)
No	14/29 (48 %)
Unsure	3/29 (10 %)

^*1*^ n/N (%).

**Table 8c:** Healthcare professional physical activity.

	N = 29^*1*^
**Physical Activity Level (IPAQ)**	
High	14/29 (48 %)
Low	2/29 (6.9 %)
Moderate	13/29 (45 %)
**Clinical exposure resulted in changes to own lifestyle choices (Regular exercise)**	
n/a	0/29 (0 %)
Strongly Disagree	3/29 (10 %)
Disagree	2/29 (6.9 %)
Neutral	6/29 (21 %)
Agree	11/29 (38 %)
Strongly Agree	7/29 (24 %)

^*1*^ n/N (%).

All HCPs reported completing some physical activity in the previous seven days, with 7 % (n = 2/29) reporting a low physical activity level. Three HCPs (10 %, n = 3/29) reported never consuming alcohol and a further 14 % (n = 4/29) report not drinking alcohol at present. Almost 76 % (n = 22/29) reported drinking more than six standard units of alcohol in one sitting at least once per month. No HCPs smoked at present and 24 % (n = 7/29) were former smokers. Many HCPs stated their clinical exposure resulted in changes to personal lifestyle choices (Table 8a–e).

**Table 8d:** Healthcare professional alcohol consumption.

	N = 29^*1*^
**Alcohol consumption**	
Not at present	4/29 (14 %)
Never	3/29 (10 %)
1–3 times per month	10/29 (34 %)
Once per week	8/29 (28 %)
2–4 times per week	3/29 (10 %)
5–6 times per week	1/29 (3.4 %)
Everyday	0/29 (0 %)
**Frequency of consumingsix or more standard unitsonone occasion**	
n/a	6/29 (21 %)
Never	11/29 (38 %)
1–3 times per month	11/29 (38 %)
Once per week	1/29 (3.4 %)
2–4 times per week	0/29 (0 %)
5–6 times per week	0/29 (0 %)
Everyday	0/29 (0 %)
**Clinical exposure resulted in changes toown lifestyle choices (Reduced alcohol intake)**	
n/a	4/29 (14 %)
Strongly Disagree	4/29 (14 %)
Disagree	3/29 (10 %)
Neutral	3/29 (10 %)
Agree	12/29 (41 %)
Strongly Agree	3/29 (10 %)

^*1*^ n/N (%).

**Table 8e:** Healthcare professional smoking status.

	N = 29^*1*^
**Smoking status**	
Never Smoked	22/29 (76 %)
Former Smoker	7/29 (24 %)
Current Smoker	0/29 (0 %)
**Current smokers doing any of the following**	
n/a	29/29 (100 %)
Trying to quit	0/29 (0 %)
Actively trying to quit	0/29 (0 %)
Not thinking of quitting	0/29 (0 %)
Thinking but not planning	0/29 (0 %)
**Clinicalexposure resulted in changes toown lifestyle choices (Smoking cessation)**	
n/a	16/29 (55 %)
Strongly Disagree	3/29 (10 %)
Disagree	0/29 (0 %)
Neutral	0/29 (0 %)
Agree	6/29 (21 %)
Strongly Agree	4/29 (14 %)

^*1*^ n/N (%).

### 3.4 Healthcare professional awareness of risk factors and service acceptability

**Fig. 8:**
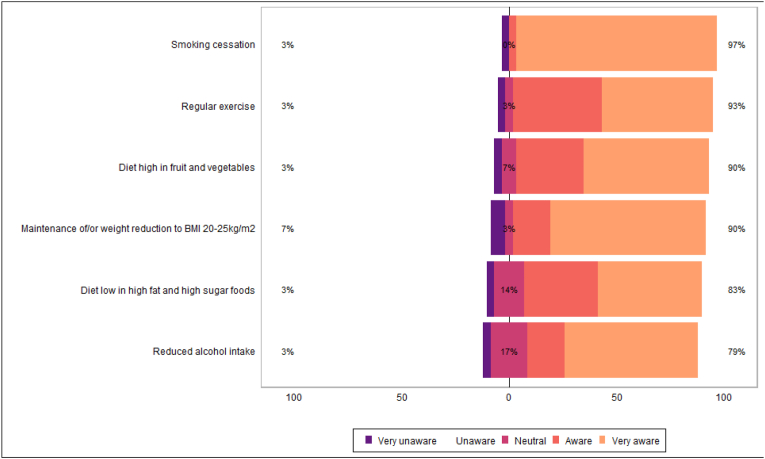
Healthcare professional awareness of lifestyle factors role in cancer development.

**Fig. 9:**
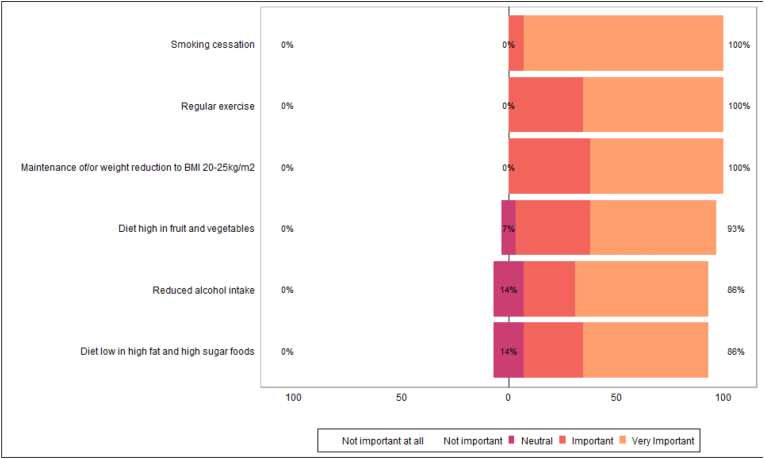
Healthcare professional beliefs on the importance of lifestyle factors in secondary cancer prevention.

Many HCPs reported awareness of the importance of modifiable lifestyle risk factors in cancer development. Most HCPs agreed that all modifiable lifestyle factors presented were important for secondary cancer prevention (Table 9, Table 10, Fig. 8, Fig. 9).

**Table 9:** Healthcare professional awareness of lifestyle factors role in cancer development.

	N = 29^*1*^
**Maintenance of/or weight reduction to BMI 20**–**25kg/m2**	
n/a	0/29 (0 %)
Very unaware	2/29 (6.9 %)
Unaware	0/29 (0 %)
Neutral	1/29 (3.4 %)
Aware	5/29 (17 %)
Very aware	21/29 (72 %)
**Diet high in fruit and vegetables**	
n/a	0/29 (0 %)
Very unaware	1/29 (3.4 %)
Unaware	0/29 (0 %)
Neutral	2/29 (6.9 %)
Aware	9/29 (31 %)
Very aware	17/29 (59 %)
**Diet low in high fat and high sugar foods**	
n/a	0/29 (0 %)
Very unaware	1/29 (3.4 %)
Unaware	0/29 (0 %)
Neutral	4/29 (14 %)
Aware	10/29 (34 %)
Very aware	14/29 (48 %)
**Regular exercise**	
n/a	0/29 (0 %)
Very unaware	1/29 (3.4 %)
Unaware	0/29 (0 %)
Neutral	1/29 (3.4 %)
Aware	12/29 (41 %)
Very aware	15/29 (52 %)
**Reduced alcohol intake**	
n/a	0/29 (0 %)
Very unaware	1/29 (3.4 %)
Unaware	0/29 (0 %)
Neutral	5/29 (17 %)
Aware	5/29 (17 %)
Very aware	18/29 (62 %)
**Smoking cessation**	
n/a	0/29 (0 %)
Very unaware	1/29 (3.4 %)
Unaware	0/29 (0 %)
Neutral	0/29 (0 %)
Aware	1/29 (3.4 %)
Very aware	27/29 (93 %)

^*1*^ n/N (%).

**Table 10:** Healthcare professional beliefs on the importance of lifestyle factors in secondary cancer prevention.

	N = 29^*1*^
**Maintenance of/or weight reduction to BMI 20**–**25kg/m2**	
n/a	0/29 (0 %)
Not important at all	0/29 (0 %)
Not important	0/29 (0 %)
Neutral	1/29 (3.4 %)
Important	12/29 (41 %)
Very Important	16/29 (55 %)
**Diet high in fruit and vegetables**	
n/a	0/29 (0 %)
Not important at all	0/29 (0 %)
Not important	0/29 (0 %)
Neutral	2/29 (6.9 %)
Important	12/29 (41 %)
Very Important	15/29 (52 %)
**Diet low in high fat and high sugar foods**	
n/a	0/29 (0 %)
Not important at all	0/29 (0 %)
Not important	0/29 (0 %)
Neutral	4/29 (14 %)
Important	12/29 (41 %)
Very Important	13/29 (45 %)
**Regular exercise**	
n/a	0/29 (0 %)
Not important at all	0/29 (0 %)
Not important	0/29 (0 %)
Neutral	2/29 (6.9 %)
Important	9/29 (31 %)
Very Important	18/29 (62 %)
**Reduced alcohol intake**	
n/a	0/29 (0 %)
Not important at all	0/29 (0 %)
Not important	0/29 (0 %)
Neutral	4/29 (14 %)
Important	7/29 (24 %)
Very Important	18/29 (62 %)
**Smoking cessation**	
n/a	0/29 (0 %)
Not important at all	0/29 (0 %)
Not important	0/29 (0 %)
Neutral	0/29 (0 %)
Important	2/29 (6.9 %)
Very Important	27/29 (93 %)

^*1*^ n/N (%).

HCPs agreed that current secondary risk reduction services were insufficient for breast cancer survivors and over 80 % agreed that services, if available, would be successful (Table 11, Table 12). Almost 90 % of HCPs were willing to refer to all proposed services if provided and over three quarters agreed that the services were likely to be effective (Table 13, Table 14).

**Table 11:** Healthcare professional perspective on sufficiency of current provision of secondary risk reduction services.

	N = 29^*1*^
**Dietetic led weight gain prevention programs**	
n/a	0/29 (0 %)
Strongly Disagree	8/29 (28 %)
Disagree	12/29 (41 %)
Neutral	6/29 (21 %)
Agree	3/29 (10 %)
Strongly Agree	0/29 (0 %)
**Dietetic led dietary education programs**	
n/a	0/29 (0 %)
Strongly Disagree	9/29 (31 %)
Disagree	10/29 (34 %)
Neutral	9/29 (31 %)
Agree	1/29 (3.4 %)
Strongly Agree	0/29 (0 %)
**Physiotherapy led physical exercise education**	
n/a	0/29 (0 %)
Strongly Disagree	7/29 (24 %)
Disagree	13/29 (45 %)
Neutral	6/29 (21 %)
Agree	2/29 (6.9 %)
Strongly Agree	1/29 (3.4 %)
**Healthcare professional led alcohol reduction service**	
n/a	0/29 (0 %)
Strongly Disagree	9/29 (31 %)
Disagree	11/29 (38 %)
Neutral	5/29 (17 %)
Agree	3/29 (10 %)
Strongly Agree	1/29 (3.4 %)
**Healthcare professional led smoking cessation service**	
n/a	0/29 (0 %)
Strongly Disagree	3/29 (10 %)
Disagree	7/29 (24 %)
Neutral	3/29 (10 %)
Agree	13/29 (45 %)
Strongly Agree	3/29 (10 %)

^*1*^ n/N (%).

**Table 12:** Healthcare professional perspective on success of secondary risk reduction services.

	N = 29^*1*^
**Dietetic led weight gain prevention programs**	
n/a	0/29 (0 %)
Strongly Disagree	1/29 (3.4 %)
Disagree	1/29 (3.4 %)
Neutral	1/29 (3.4 %)
Agree	15/29 (52 %)
Strongly Agree	11/29 (38 %)
**Dietetic led dietary education programs**	
n/a	0/29 (0 %)
Strongly Disagree	0/29 (0 %)
Disagree	3/29 (10 %)
Neutral	0/29 (0 %)
Agree	16/29 (55 %)
Strongly Agree	10/29 (34 %)
**Physiotherapy led physical exercise education**	
n/a	0/29 (0 %)
Strongly Disagree	0/29 (0 %)
Disagree	1/29 (3.4 %)
Neutral	2/29 (6.9 %)
Agree	14/29 (48 %)
Strongly Agree	12/29 (41 %)
**Healthcare professional led alcohol reduction service**	
n/a	0/29 (0 %)
Strongly Disagree	1/29 (3.4 %)
Disagree	2/29 (6.9 %)
Neutral	2/29 (6.9 %)
Agree	14/29 (48 %)
Strongly Agree	10/29 (34 %)
**Healthcare professional led smoking cessation service**	
n/a	0/29 (0 %)
Strongly Disagree	0/29 (0 %)
Disagree	3/29 (10 %)
Neutral	0/29 (0 %)
Agree	14/29 (48 %)
Strongly Agree	12/29 (41 %)

^*1*^ n/N (%).

**Table 13:** Healthcare professional likeliness to refer to secondary risk reduction services.

	N = 29^*1*^
**Dietetic led weight gain prevention programs**	
n/a	0/29 (0 %)
Very unlikely	0/29 (0 %)
Unlikely	0/29 (0 %)
Neutral	1/29 (3.4 %)
Likely	11/29 (38 %)
Very Likely	17/29 (59 %)
**Dietetic led dietary education programs**	
n/a	0/29 (0 %)
Very unlikely	0/29 (0 %)
Unlikely	0/29 (0 %)
Neutral	1/29 (3.4 %)
Likely	11/29 (38 %)
Very Likely	17/29 (59 %)
**Physiotherapy led physical exercise education**	
n/a	0/29 (0 %)
Very unlikely	0/29 (0 %)
Unlikely	0/29 (0 %)
Neutral	0/29 (0 %)
Likely	11/29 (38 %)
Very Likely	18/29 (62 %)
**Healthcare professional led alcohol reduction service**	
n/a	0/29 (0 %)
Very unlikely	0/29 (0 %)
Unlikely	1/29 (3.4 %)
Neutral	2/29 (6.9 %)
Likely	8/29 (28 %)
Very Likely	18/29 (62 %)
**Healthcare professional led smoking cessation service**	
n/a	0/29 (0 %)
Very unlikely	0/29 (0 %)
Unlikely	0/29 (0 %)
Neutral	2/29 (6.9 %)
Likely	7/29 (24 %)
Very Likely	20/29 (69 %)

^*1*^ n/N (%).

**Table 14:** Healthcare professional perception of effectiveness of secondary risk reduction services.

	N = 29^*1*^
**Dietetic led weight gain prevention programs**	
n/a	0/29 (0 %)
Very unlikely	0/29 (0 %)
Unlikely	0/29 (0 %)
Neutral	4/29 (14 %)
Likely	14/29 (48 %)
Very Likely	11/29 (38 %)
**Dietetic led dietary education programs**	
n/a	0/29 (0 %)
Very unlikely	0/29 (0 %)
Unlikely	0/29 (0 %)
Neutral	2/29 (6.9 %)
Likely	20/29 (69 %)
Very Likely	7/29 (24 %)
**Physiotherapy led physical exercise education**	
n/a	1/29 (3.4 %)
Very unlikely	0/29 (0 %)
Unlikely	1/29 (3.4 %)
Neutral	1/29 (3.4 %)
Likely	15/29 (52 %)
Very Likely	11/29 (38 %)
**Healthcare professional led alcohol reduction service**	
n/a	0/29 (0 %)
Very unlikely	1/29 (3.4 %)
Unlikely	3/29 (10 %)
Neutral	3/29 (10 %)
Likely	13/29 (45 %)
Very Likely	9/29 (31 %)
**Healthcare professional led smoking cessation service**	
n/a	0/29 (0 %)
Very unlikely	0/29 (0 %)
Unlikely	4/29 (14 %)
Neutral	4/29 (14 %)
Likely	12/29 (41 %)
Very Likely	9/29 (31 %)

^*1*^ n/N (%).

## 4 Discussion

Increased BMI, poor quality diet, low physical activity, increased alcohol, and smoking are modifiable lifestyle risk factors in primary and secondary cancer development [6,29]. Both patients and HCPs surveyed in our study met several at-risk criteria. This study identified a high awareness among breast cancer survivors and oncology HCPs of the role that modifiable lifestyle risk factors play in cancer development. In addition, both cohorts reported acceptability of secondary risk reduction services, if more widely available within Irish breast cancer services.

Obesity rates in Ireland have increased over the last decade [29]. BMI measurement correlates well with the amount of adiposity in most of the general population; however, it has limitations. Nevertheless, it provides essential epidemiological insights into the impact that increased adiposity has on chronic disease development, including cancer [30]. A large proportion of our patient cohort self-reported a BMI in the overweight or obese category and over one-third were trying to lose weight. These results align with the Healthy Ireland Survey 2019, which demonstrated that 60 % of the Irish population had a self-reported BMI of greater than 25kg/m2, and 34 % were actively trying to lose weight however weight management is complex [31]. Multiple randomised control trials, many employing the social cognitive theory of behaviour change have demonstrated the benefits of weight loss interventions in breast cancer survivors [[32], [33], [34], [35], [36], [37], [38], [39], [40], [41]]. Assessment of impact on survival and disease recurrence in breast cancer is ongoing; however, interim results of one American study demonstrated a potential clinically relevant 29 % reduction in disease recurrence [35].

Healthy eating is an essential aspect of weight management and chronic disease prevention. Nearly one-third of patients did not meet the recommended minimum of five portions of fruit and vegetable intake daily and had regular consumption of high-fat, sugar, and salt foods. Positively, over 40 % of patients had made improvements to their dietary habits however, high levels of stress eating was reported which can be barrier to maintaining a healthy diet in breast cancer [42].

Physical activity has been shown to improve fatigue, cardiorespiratory fitness, physical functioning, and quality of life for breast cancer survivors [43]. In addition, breast cancer survivors who are more physically active have a reduced disease recurrence and increased survival [44]. Irish breast cancer survivors have identified that advice on diet, exercise, and a healthy lifestyle is essential to them in their survivorship journey [15]. Physical activity levels in our patient cohort were reported as moderate to high, furthermore, a large proportion of patients had increased exercise since diagnosis. Despite this, studies have shown that a lower percentage of cancer survivors meet physical activity guidelines in comparison to age-matched individuals without cancer [45]. Disease-related fatigue and lack of clear guidelines can limit patient engagement with exercise [46]. Breast cancer survivors are keen to receive information on exercise however they have additional disease-related burdens; therefore, a specialist service providing tailored education and support would be beneficial.

Five per cent of our patient cohort reported smoking; however, 88 % were thinking about or actively planning to quit and the prevalence of current smokers was lower than the 14.1 % reported in similar studies [45]. Of note, 42.8 % of patients were former smokers, and 80 % had given up more than five years ago. The number of former smokers in our patient cohort is higher than the sex and age-matched group of smokers in the TILDA report 2011 (31 %), indicating that smoking may have been a predisposing risk factor [47]. In 2004, Ireland became the first country worldwide to introduce a smoke-free workplace law [48]. Over the last two decades, the changes in smoking practices across Ireland demonstrate that the Irish population are amenable to lifestyle change. The annual ‘Healthy Ireland Survey 2019’ results showed a further reduction in daily smoking from 23 % to 16 % in the previous five years, indicating that progress continues to be made [49]. A recent French review demonstrated that one-third of breast cancer patients who were provided information on smoking post-diagnosis decided to quit with the primary motivating factor reported as the risk of recurrence [50].

Alcohol consumption has a moderate risk in the development of cancer [51]. An Irish study demonstrated low levels of awareness of alcohol as a risk factor in cancer development; however, there was a high level of awareness reported amongst our cohort [52]. Despite this awareness, almost 40 % of our cohort reported consumption of more than six units of alcohol in one sitting. This pattern of alcohol consumption carries with it a high risk of health issues, particularly in breast cancer, where there is an increased risk associated with heavy episodic drinking amongst moderate lifetime drinkers [53]. Alcohol consumption in the Irish population decreases from the age of 50–80 years of age; however, given breast cancer in Ireland is most commonly diagnosed after the age of 50, education is required earlier to ameliorate the risk associated with this behaviour [47]. Furthermore, awareness of alcohol as a risk factor in breast cancer has been associated with the knowledge necessary for behaviour change [54]. In our patient cohort, over one quarter had made changes to alcohol consumption since diagnosis. Despite the substantial data to support alcohol reduction, only 1.2 % of patients in our cohort had received advice on reducing alcohol intake, an essential finding for clinician education and health promotion. Breast cancer diagnosis or screening is a pivotal time for encouraging alcohol reduction; however, more tailored health promotion and advice-giving are required. A large proportion of our patient cohort had made changes to their lifestyle choices since diagnosis. Research demonstrates that awareness of risk factors and intention to prevent further illness after breast cancer diagnosis was associated with a willingness to change lifestyle behaviours [50,[54], [55], [56]].

The high rate of modifiable risk factors in our cohort aligns with findings from other studies [57,58]. In healthcare, this prevalence in the context of a life-threatening cancer diagnosis would be considered a highly relevant teachable moment where health behaviour change can be stimulated by patient action [59]. However, these studies have also highlighted variable degrees of behaviour modification following breast cancer diagnosis and the challenges in increasing both motivation for, and adherence to physical activity programs [[60], [61], [62]]. While many patients reported an intention to engage in dietary and exercise programmes, many HCPs surveyed believed that the provision of such services in Ireland was insufficient. Although extensive research highlights the benefits of exercise in preventing and managing chronic diseases, access to exercise education remains low. Many HCPs are keen to provide advice on physical activity; however, the lack of structured support services leaves them with limited time to provide an exercise prescription whilst focusing on other clinical priorities [63]. This is compounded by the reality that exercise and lifestyle medicine is underemphasised in medical training [64,65]. The timing of lifestyle modification advice and intervention is also pivotal [66]. Patients have voiced an openness to receiving lifestyle advice at the time of diagnosis; however, they favoured engaged at a later stage [67]. These are significant findings for secondary risk reduction service development.

To our knowledge, this is the first study to establish the proportion of Irish breast survivors who meet all at-risk lifestyle criteria outlined. Limitations include collection restricted to two clinical sites and a short data-gathering period. The use of self-reported questionnaires is also a limitation, with studies demonstrating an underestimation of anthropometric data and an overestimation of physical activity; therefore, objective measurements in subsequent studies would be favourable [[68], [69], [70]]. Sleep and stress are important factors to consider in lifestyle behaviour modification, and any future studies should include an analysis of these factors. Based on the results of this study the authors have conducted a subsequent survey of breast cancer survivors assessing the optimum integration of the teachable moment of behaviour modification in their care [71]. Further research is also required to understand oncology HCPs perspectives on secondary prevention education.

In summary, the focus in breast cancer care is on pharmacological prescribing; however, the weight of evidence supports the importance of integrating lifestyle interventions in parallel for this cohort [72]. Our study demonstrates that a proportion of patients met at-risk criteria for modifiable lifestyle risk factors, addressing them could improve their quality of life and breast cancer outcomes. Structuring diagnosis-specific secondary risk reduction services in our health services needs to be prioritised. In the interim, patients should be screened for at-risk criteria and receive counselling on the importance of lifestyle modification as part of their breast cancer treatment plan. These measures have the potential to improve their cancer-related morbidity and mortality and may reduce the development or worsening of other chronic diseases.

## CRediT authorship contribution statement

**Clara Steele:** Writing – review & editing, Writing – original draft, Formal analysis, Data curation, Conceptualization. **Janas M. Harrington:** Writing – review & editing, Conceptualization. **Seamus O'Reilly:** Writing – review & editing, Writing – original draft, Formal analysis, Data curation, Conceptualization.

## Declaration of competing interest

The authors declare there are no financial interests/personal relationships which may be considered as potential competing interests.
