# Supplementary material for: Awareness of modifiable lifestyle risk factors and acceptability of secondary risk reduction services amongst Irish breast cancer survivors and oncology healthcare professionals
Source: Breast. 2025 Aug 19;84:104561. doi: 10.1016/j.breast.2025.104561 (PMC12554229; doi:10.1016/j.breast.2025.104561)
Supplement: Multimedia component 1 [file mmc1.docx]

**Questionnaire – Patients**

Participant Information

Section 1 - Demographics

1. **What age are you? (years)**

18-30 31-40 41-50 51-60

61-70 71-80 81+

1. **What is your gender?**

Male Female Prefer not to say

1. **What is the highest level of education you have completed to date?**

Some primary (not complete) Primary or equivalent

Inter/junior cert or equivalent  Leaving cert or equivalent

Diploma/cert  Primary university degree

Postgraduate/higher degree

1. **What is your diagnosis?**

Breast Cancer (first cancer diagnosis) Breast Cancer (second cancer diagnosis)

Other diagnosis

1. **What year were you first diagnosed with breast cancer?** __________
2. **Have you been diagnosed with more than one cancer?**

Yes No If yes, please specify the other diagnosis __________

1. **Treatment received (please tick all that are applicable)**

Surgery Radiotherapy Chemotherapy

Hormone Treatment Not sure None

Section 2 - Anthropometric measurements

1. **What is your approximate weight (**in kilograms or stone and pounds) ________
2. **What is your approximate height** (in metres or feet and inches) ________
3. **Have you experienced involuntary weight loss, in the last 3 months?**

Yes No Unsure

If yes, how much weight have you lost?

~ 1 to 3kg (2.2-6.6lbs) ~ 3kg (6.6 lbs) More than 3kg (6.6lbs)

Other, please specify ____

1. **Has your clothes size decreased since your diagnosis?**

Yes No Unsure

If so, by how much? -1 size -2 sizes -3 sizes

1. **Have you experienced involuntary weight gain, in the last 3 months?**

Yes No Unsure

If yes, how much weight have you gained?

~ 1 to 3kg (2.2-6.6lbs) ~ 3kg (6.6 lbs) More than 3kg (6.6lbs)

Other, please specify ____

1. **Have you gained weight since your diagnosis?**

Yes No Unsure

If yes, how much weight have you gained?

~ 1 to 3kg (2.2-6.6lbs) ~ 3kg (6.6 lbs) More than 3kg (6.6lbs)

Other, please specify ____

1. **Has your clothes size increased since your diagnosis?**

Yes No Unsure

If so, by how much? +1 size +2 sizes +3 sizes

1. **Since your diagnosis, have you been trying to lose weight?**

Yes No Unsure

If yes, what was the reason for this _______

1. **Are you trying to actively lose weight at present?**

Yes No Unsure

1. **In the past 12 months has a doctor, nurse or other health professional advised you to lose, maintain, or gain weight?**

Yes No Unsure

Section 3 - Dietary intake

1. **Do you follow any of the following diets? (Tick all that apply)**

Vegetarian Vegan Gluten Free

Diabetic Weight reducing Cholesterol lowering

Other, please specify ______

1. **How many times per week do you eat fried food?**

Everyday 5-6 times per week 2-4 times per week

Once per week 1-3 times per month Never

1. **How often do you add salt to food while cooking?**

Everyday 5-6 times per week 2-4 times per week

Once per week 1-3 times per month Never

1. **How often do you add salt to food at the table?**

Everyday 5-6 times per week 2-4 times per week

Once per week 1-3 times per month Never

1. **How often do you eat foods that are high in sugar?**

*An example of high sugar food includes - cakes, biscuits, chocolate, sweets, pastries, crisps, fizzy drinks.*

Everyday 5-6 times per week 2-4 times per week

Once per week 1-3 times per month Never

1. **Approximately, how many portions of fruit do you consume each day?**

*An example of one portion is the equivalent of ~80g of fresh fruit or 30g of dried fruit. This is approximately equal to -*

- *One medium apple or banana, one canned peach*
- *One handful of strawberries or raspberries*
- *Two satsumas, plums or kiwis*

None 1-2 portions 3-4 portions

5 portions More than 5 portions

1. **Approximately, how many portions of vegetables do you consume each day?**

*An example of one portion is the equivalent of ~80g of vegetables. This is approximately equal to -*

- *Three tablespoons of carrots, sweetcorn, peas, beans, lentils, chickpeas*
- *One handful of cherry tomatoes or ½ an avocado*
- *One bowl of salad leaves or four heaped tablespoons of cooked spinach*

None 1-2 portions 3-4 portions

5 portions More than 5 portions

1. **Do you feel that you have eaten as a way to deal with stress since your diagnosis?**

Yes No Unsure

If so, how often? Once per month Once per week Daily

1. **Has your food intake declined over the last 3 months?**

Yes No Unsure

1. **In the past 12 months has a doctor, nurse or other health professional discussed ways of changing your diet with you?**

Yes No Unsure

1. **Since your diagnosis, have you made any changes to your diet?**

Yes No Unsure

If yes, what was the reason for this _________________________________________________

Section 4 - Physical Activity

We are interested in finding out about the kinds of physical activities that people do as part of their everyday lives. The questions will ask you about the time you spent being physically active in the **last 7 days**. Please answer each question even if you do not consider yourself to be an active person. Please think about the activities you do at work, as part of your house and yard work, to get from place to place, and in your spare time for recreation, exercise or sport.

Think about all the **vigorous** activities that you did in the **last 7 days**. **Vigorous** physical activities refer to activities that take hard physical effort and make you breathe much harder than normal. Think *only* about those physical activities that you did for at least 10 minutes at a time.

1. **During the last 7 days, on how many days did you do vigorous physical activities like heavy lifting, digging, aerobics, or fast bicycling?**

______ days per week

No vigorous physical activity **-** *Skip to question 31*

1. **How much time did you usually spend doing vigorous physical activities on one of those days?**

______ hours per day

______ minutes per day

Don’t know/unsure

Think about all the **moderate** activities that you did in the **last 7 days**. **Moderate** activities refer to activities that take moderate physical effort and make you breathe somewhat harder than normal. Think only about those physical activities that you did for at least 10 minutes at a time.

1. **During the last 7 days, on how many days did you do moderate physical activities like carrying light loads, bicycling at a regular pace, or doubles tennis? Do not include walking.**

______ days per week

No moderate physical activity **-** *Skip to question 33*

1. **How much time did you usually spend doing moderate physical activities on one of those days?**

______ hours per day

______ minutes per day

Don’t know/unsure

Think about the time you spent **walking** in the **last 7 days**. This includes at work and at home, walking to travel from place to place, and any other walking that you have done solely for recreation, sport, exercise, or leisure.

1. **During the last 7 days, on how many days did you walk for at least 10 minutes at a time?**

______ days per week

No walking - *Skip to question 35*

1. **How much time did you usually spend walking on one of those days?**

______ hours per day

______ minutes per day

Don’t know/unsure

The last question is about the time you spent **sitting** on weekdays during the **last 7 days**. Include time spent at work, at home, while doing course work and during leisure time. This may include time spent sitting at a desk, visiting friends, reading, or sitting or lying down to watch television.

1. **How much time did you usually spend sitting on a week day?**

______ hours per day

______ minutes per day

Don’t know/unsure

1. **Since your diagnosis, have you made any changes to the amount of physical activity that you do?**

Yes No Unsure

If yes, what was the reason for this ________________________________________________

Section 5 - Alcohol consumption

1. **Do you consume alcohol?**

Everyday 5-6 times per week 2-4 times per week

Once per week 1-3 times per month

Never - *If you have never drank alcohol - please skip to Section 6*

Not at present - *If you do not currently drink alcohol – please skip to Question 38*

1. **If you drank alcohol in the past, about how long has it been since you last drank?**

Less than one year 5-10 years ago

1-5 years ago >10 years ago

1. **If you currently drink, how often do you have six or more standard units on one occasion?**

*If you do not currently drink alcohol – please skip to Section 6*

*An example of one standard unit is equal to:*

- *One pub measure of spirits (35.5ml)*
- *One small glass of wine (12.5% volume)*
- *One half pint of normal beer*

Everyday 5-6 times per week 2-4 times per week

Once per week 1-3 times per month Never

1. **In the past 12 months has a doctor, nurse or other health professional discussed ways of reducing your alcohol with you**?

Yes No Unsure

1. **Since your diagnosis, have you made any changes to amount of alcohol you consume?**

Yes No Unsure

If yes, what was the reason for this _________________________________________________

Section 6 - Smoking status

1. **Have you yourself smoked at least 100 cigarettes in your entire life? *(5 packs = 100 cigarettes)***

Yes No Unsure

1. **Do you currently smoke or are you a former smoker?**

Current Former Never smoked

*If you have never smoked – please skip to Section 7*

1. **If you are a former smoker, about how long has it been since you last smoked?**

Less than one year 5-10 years ago

1-5 years ago >10 years ago

1. **If you are a current smoker, do you smoke every day or some days?**

Everyday Some days per week

*If you do not currently smoke – please skip to Section 7*

1. **Are you currently doing any of the following listed here?**

Trying to quit Actively trying to quit

Not thinking of quitting Thinking about it, but not planning

1. **In the past 12 months has a doctor, nurse or other health professional discussed ways of giving up smoking with you?**

Yes No Unsure

1. **Since your diagnosis, have you made changes to the amount you smoke?**

Yes No Unsure

If yes, what was the reason for this _________________________________________________

_______________________________________________________________________________

Section 7 - Secondary prevention strategies

This is the final section of the survey

- *Healthy body mass index (BMI) is a measure of your weight for your height. A normal BMI is defined as a measurement between 20-25kg/m^2^.*
- *Diet high fruit and vegetables is defined as consumption of five or more portions of fruit and/or vegetables per day.*
- *High fat/high sugar foods include cakes, biscuits, chocolate, sweets, pastries, crisps, fizzy drinks.*
- *Regular exercise defined is as 150mins of activity per week or 30mins five times per week.*
- *Safe upper limit of alcohol is defined as less than 14 units of alcohol per week.*

1. **How aware are you on the importance of the following lifestyle factors and the role they play in the development of cancer?**

*n/a = not applicable, 1 = very unaware, 2 = unaware, 3 = neutral, 4 = aware, 5 = very aware*

Maintenance of/or weight reduction to a normal BMI n/a 1 2 3 4 5

Increased fruit and vegetable consumption n/a 1 2 3 4 5

Reduced high fat/high sugar food consumption n/a 1 2 3 4 5

Regular exercise n/a 1 2 3 4 5

Reduced alcohol intake n/a 1 2 3 4 5

Smoking cessation n/a 1 2 3 4 5

1. **How important do you think the following lifestyle factors are in preventing secondary cancers?**

*1 = not important at all, 2 =not important, 3 = neutral, 4 = important, 5 = very important*

Normal body mass index 1 2 3 4 5

Diet high in fruit and vegetables 1 2 3 4 5

Diet low in high fat/high sugar foods 1 2 3 4 5

Regular exercise 1 2 3 4 5

Reduced alcohol intake 1 2 3 4 5

Quitting smoking 1 2 3 4 5

1. **Would it be important for you to have access to the following services?**

n/a = not applicable*, 1= not important at all, 2 =not important, 3 = neutral, 4 = important, 5 = very important*

Dietetic led weight gain prevention programs n/a 1 2 3 4 5

Dietetic led dietary education programs n/a 1 2 3 4 5

Physiotherapy led physical exercise education n/a 1 2 3 4 5

Healthcare professional led alcohol reduction n/a 1 2 3 4 5

Healthcare professional led smoking cessation n/a 1 2 3 4 5

1. **If the following services were available, how likely would it be for you to use them?**

*n/a = not applicable, 1 = very unlikely, 2 = unlikely, 3 = neutral, 4 = likely, 5 = very likely*

Dietetic led weight gain prevention programs n/a 1 2 3 4 5

Dietetic led dietary education programs n/a 1 2 3 4 5

Physiotherapy led physical exercise education n/a 1 2 3 4 5

Healthcare professional led alcohol reduction n/a 1 2 3 4 5

Healthcare professional led smoking cessation n/a 1 2 3 4 5

1. **What do you think would be the most reliable source of advice regarding lifestyle change? (tick all that apply)**

General Practitioner Oncology Doctor Oncology Nurse

Online Websites Other healthcare professional i.e., dietitian or physiotherapist

Support services i.e. Arc House Other, please specify ______________________

1. **Do you have any suggestions for other diet and lifestyle related services you would like access to?**

_________________________________________________________________________________

_________________________________________________________________________________

1. **Is there anything else you wish to add or comment on?**

_________________________________________________________________________________

_________________________________________________________________________________

Thank you for taking the time to complete our questionnaire.

**Questionnaire – Clinicians**

Participant Information

Section 1 - Demographics

1. **Age (years)**

18-30 31-40 41-50 51-60

61-70 71-80 81+

1. **Gender**

Male Female Prefer not to say

1. **Occupation**

Clinical Nurse Specialist

Registrar

Specialist Registrar

Consultant

Other

1. **Number of years of oncology experience**

<1 1-4 5-9 10-19 20+

1. **Have you completed any postgraduate training in secondary prevention for example, nutrition or exercise medicine?**

Yes No Prefer not to say

If you said yes above, what area? (tick all that apply)

Nutrition Sports/Exercise Medicine

Behavioural change Other, please specify ________________

Section 2 - Anthropometric measurements

1. **What is your approximate weight** (in kilograms or stone and pounds) ________
2. **What is your approximate height** (in metres or feet and inches) ________
3. **Are you trying to actively lose weight at present?**

Yes No Unsure

Section 3 - Dietary intake

1. **Do you follow any of the following diets? (Tick all that apply)**

Vegetarian Vegan Gluten Free

Diabetic Weight reducing Cholesterol lowering

Other, please specify ______

1. **How many times per week do you eat fried food?**

Everyday 5-6 times per week 2-4 times per week

Once per week 1-3 times per month Never

1. **How often do you add salt to food while cooking?**

Everyday 5-6 times per week 2-4 times per week

Once per week 1-3 times per month Never

1. **How often do you add salt to food at the table?**

Everyday 5-6 times per week 2-4 times per week

Once per week 1-3 times per month Never

1. **How often do you eat foods that are high in sugar?**

*An example of a high sugar containing food includes cakes, biscuits, chocolate, sweets, pastries, crisps, fizzy drinks.*

Everyday 5-6 times per week 2-4 times per week

Once per week 1-3 times per month Never

1. **Approximately, how many portions of fruit do you consume each day?**

*An example of one portion is the equivalent of ~80g of fresh fruit or 30g of dried fruit. This is approximately equal to -*

- *One medium apple or banana, one canned peach*
- *One handful of strawberries or raspberries*
- *Two satsumas, plums or kiwis*

None 1-2 portions 3-4 portions

5 portions More than 5 portions

1. **Approximately, how many portions of vegetables do you consume each day?**

*An example of one portion is the equivalent of ~80g of vegetables. This is approximately equal to -*

- *Three tablespoons of carrots, sweetcorn, peas, beans, lentils, chickpeas*
- *One handful of cherry tomatoes or ½ an avocado*
- *One bowl of salad leaves or four heaped tablespoons of cooked spinach*

None 1-2 portions 3-4 portions

5 portions More than 5 portions

1. **Do you feel that you eat as a way to deal with stress?**

Yes No Unsure

If so, how often? Once per month Once per week Daily

Section 4 - Physical Activity

We are interested in finding out about the kinds of physical activities that people do as part of their everyday lives. The questions will ask you about the time you spent being physically active in the **last 7 days**. Please answer each question even if you do not consider yourself to be an active person. Please think about the activities you do at work, as part of your house and yard work, to get from place to place, and in your spare time for recreation, exercise or sport.

Think about all the **vigorous** activities that you did in the **last 7 days**. **Vigorous** physical activities refer to activities that take hard physical effort and make you breathe much harder than normal. Think *only* about those physical activities that you did for at least 10 minutes at a time.

1. **During the last 7 days, on how many days did you do vigorous physical activities like heavy lifting, digging, aerobics, or fast bicycling?**

______ days per week

No vigorous physical activity - *Skip to question 19*

1. **How much time did you usually spend doing vigorous physical activities on one of those days?**

______ hours per day

______ minutes per day

Don’t know/unsure

Think about all the **moderate** activities that you did in the **last 7 days**. **Moderate** activities refer to activities that take moderate physical effort and make you breathe somewhat harder than normal. Think only about those physical activities that you did for at least 10 minutes at a time.

1. **During the last 7 days, on how many days did you do moderate physical activities like carrying light loads, bicycling at a regular pace, or doubles tennis? Do not include walking.**

______ days per week

No moderate physical activity - *Skip to question 21*

1. **How much time did you usually spend doing moderate physical activities on one of those days?**

______ hours per day

______ minutes per day

Don’t know/unsure

Think about the time you spent **walking** in the **last 7 days**. This includes at work and at home, walking to travel from place to place, and any other walking that you have done solely for recreation, sport, exercise, or leisure.

1. **During the last 7 days, on how many days did you walk for at least 10 minutes at a time?**

______ days per week

No vigorous physical activity - *Skip to question 23*

1. **How much time did you usually spend walking on one of those days?**

______ hours per day

______ minutes per day

Don’t know/unsure

The last question is about the time you spent **sitting** on weekdays during the **last 7 days**. Include time spent at work, at home, while doing course work and during leisure time. This may include time spent sitting at a desk, visiting friends, reading, or sitting or lying down to watch television.

1. **How much time did you usually spend sitting on one of those days?**

______ hours per day

______ minutes per day

Don’t know/unsure

Section 5 - Alcohol consumption

1. **Do you consume alcohol?**

Everyday 5-6 times per week 2-4 times per week

Once per week 1-3 times per month

Never - *If you have never drank alcohol - please skip to Section 6*

Not at present - *If you do not currently drink alcohol – please skip to Question 25*

1. **If you drank alcohol in the past, about how long has it been since you last drank?**

Less than one year 5-10 years ago

1-5 years ago >10 years ago

1. **If you currently drink alcohol, how often do you have six or more standard units on one occasion?**

*If you do not currently drink alcohol – please skip to Section 6*

*An example of one standard unit is equal to:*

- *One pub measure of spirits (35.5ml)*
- *One small glass of wine (12.5% volume)*
- *One half pint of normal beer*

Everyday 5-6 times per week 2-4 times per week

Once per week 1-3 times per month Never

1. **In the past 12 months has a doctor, nurse or other health professional discussed ways of reducing your alcohol with you?**

Yes No Unsure

Section 6 - Smoking status

1. **Have you yourself smoked at least 100 cigarettes in your entire life?**

*(5 packs = 100 cigarettes)*

Yes No Don’t know

1. **Do you currently smoke or are you a former smoker?**

Current Former Never smoked

*If you have never smoked – please skip to Section 7*

1. **If you are a former smoker, about how long has it been since you last smoked? (now question 30)**

Less than one year 5-10 years ago

1-5 years ago >10 years ago

1. **If you are a current smoker, do you smoke every day or some days?**

Everyday Some days

*If you do not currently smoke - please skip to Section 7*

1. **Are you currently doing any of the following listed here?**

Trying to quit Actively trying to quit

Not thinking of quitting Thinking about it, but not planning

1. **In the past 12 months has a doctor, nurse or other health professional discussed ways of giving up smoking with you?**

Yes No Unsure

Section 7 - Lifestyle changes

- *Healthy body mass index (BMI) is a measure of your weight for your height. A normal BMI is defined as a measurement between 20-25kg/m^2^.*
- *Diet high fruit and vegetables is defined as consumption of five or more portions of fruit and/or vegetables per day.*
- *High fat/high sugar foods include cakes, biscuits, chocolate, sweets, pastries, crisps, fizzy drinks.*
- *Regular exercise defined is as 150mins of activity per week or 30mins five times per week.*
- *Safe upper limit of alcohol is defined as less than 14 units of alcohol per week.*

1. **Has your clinical exposure resulted in changes to your own lifestyle choices?**

*1 = strongly disagree, 2 = disagree, 3 = neither agree nor disagree, 4 = agree, 5 = strongly agree, n/a = not applicable*

Maintenance of/or weight reduction to a normal BMI 1 2 3 4 5 n/a

Increased fruit and vegetable consumption 1 2 3 4 5 n/a

Reduced high fat/high sugar food consumption 1 2 3 4 5 n/a

Regular exercise 1 2 3 4 5 n/a

Reduced alcohol intake 1 2 3 4 5 n/a

Smoking cessation 1 2 3 4 5 n/a

Section 8 - Secondary prevention strategies

This the final section of the survey.

1. **In your opinion, how important do you think that secondary cancer prevention is?**

*1 = not important at all, 2 = not important, 3 = neutral, 4 = important, 5 = very important*

1 2 3 4 5

1. **How aware are you of the importance of the following lifestyle factors and the role they play in cancer development?**

*1 = very unaware, 2 = unaware, 3 = neutral, 4 = aware, 5 = very aware*

Maintenance of/or weight reduction to BMI 20-25kg/m^2^ 1 2 3 4 5

Diet high in fruit and vegetables 1 2 3 4 5

Diet low in high fat and high sugar foods 1 2 3 4 5

Regular exercise 1 2 3 4 5

Reduced alcohol intake 1 2 3 4 5

Smoking cessation 1 2 3 4 5

1. **Do you think the following lifestyle factors are important in preventing secondary cancers?**

*1 = strongly disagree, 2 = disagree, 3 = neither agree nor disagree, 4 = agree, 5 = strongly agree*

Maintenance of/or weight reduction to BMI 20-25kg/m^2^ 1 2 3 4 5

Diet high in fruit and vegetables 1 2 3 4 5

Diet low in high fat and high sugar foods 1 2 3 4 5

Regular exercise 1 2 3 4 5

Reduced alcohol intake 1 2 3 4 5

Smoking cessation 1 2 3 4 5

1. **How important would you rate the following lifestyle factors in preventing secondary cancers?**

*1 = not important at all, 2 = not important, 3 = neutral, 4 = important, 5 = very important*

Maintenance of/or weight reduction to BMI 20-25kg/m^2^ 1 2 3 4 5

Diet high in fruit and vegetables 1 2 3 4 5

Diet low in high fat and high sugar foods 1 2 3 4 5

Regular exercise 1 2 3 4 5

Reduced alcohol intake 1 2 3 4 5

Smoking cessation 1 2 3 4 5

1. **Do you feel that secondary cancer prevention is important for breast cancer patients?**

*1 = not important at all, 2 = not important, 3 = neutral, 4 = important, 5 = very important*

1 2 3 4 5

1. **Do you think that secondary cancer prevention is sufficiently emphasised in the following?**

*1 = strongly disagree, 2 = disagree, 3 = neither agree nor disagree, 4 = agree, 5 = strongly agree*

Medical Education 1 2 3 4 5

Healthcare Policy 1 2 3 4 5

Clinical Practice 1 2 3 4 5

1. **Do you think the current provision of these services for breast cancer patients is sufficient?**

*1 = strongly disagree, 2 = disagree, 3 = neither agree nor disagree, 4 = agree, 5 = strongly agree*

Dietetic led weight gain prevention programs 1 2 3 4 5

Dietetic led dietary education programs 1 2 3 4 5

Physiotherapy led physical exercise education 1 2 3 4 5

Healthcare professional led alcohol reduction service 1 2 3 4 5

Healthcare professional led smoking cessation service 1 2 3 4 5

1. **Cancer prevention services are an aspiration of the National Cancer Strategy 2017. Based on your clinical experience which of these services, if available, do you think would be successful?**

*1 = strongly disagree, 2 = disagree, 3 = neither agree nor disagree, 4 = agree, 5 = strongly agree*

Dietetic led weight gain prevention programs 1 2 3 4 5

Dietetic led dietary education programs 1 2 3 4 5

Physiotherapy led physical exercise education 1 2 3 4 5

Healthcare professional led alcohol reduction service 1 2 3 4 5

Healthcare professional led smoking cessation service 1 2 3 4 5

1. **If the following services were available to patients, how likely would it be for you to refer patients to them?**

*1 = very unlikely, 2 = unlikely, 3 = neutral, 4 = likely, 5 = very likely*

Dietetic led weight gain prevention programs 1 2 3 4 5

Dietetic led dietary education programs 1 2 3 4 5

Physiotherapy led physical exercise education 1 2 3 4 5

Healthcare professional led alcohol reduction service 1 2 3 4 5

Healthcare professional led smoking cessation service 1 2 3 4 5

1. **If the following services were available to patients, do you think that these services are likely to be effective?**

*1 = very unlikely, 2 = unlikely, 3 = neutral, 4 = likely, 5 = very likely*

Dietetic led weight gain prevention programs 1 2 3 4 5

Dietetic led dietary education programs 1 2 3 4 5

Physiotherapy led physical exercise education 1 2 3 4 5

Healthcare professional led alcohol reduction service 1 2 3 4 5

Healthcare professional led smoking cessation service 1 2 3 4 5

1. **If you think any of the aforementioned services may not be effective, please explain why.**

_________________________________________________________________________________

_________________________________________________________________________________

1. **If the following resources were available to patients, what resource do you think would be most effective?**

*1 = not effective at all, 2 = not effective, 3 = neutral, 4 = effective, 5 = very effective*

Healthcare professional led websites 1 2 3 4 5

Healthcare professional led e-learning 1 2 3 4 5

Healthcare professional led online education groups 1 2 3 4 5

Healthcare professional written information booklet 1 2 3 4 5

1. **Do you have any suggestions for other diet and lifestyle related services or resources, that may be of benefit to patients? Please list below.**

_________________________________________________________________________________

_________________________________________________________________________________

1. **Is there anything else you wish to add or comment on?**

_________________________________________________________________________________

_________________________________________________________________________________

Thank you for taking the time to complete our questionnaire.
